# Supplementary material for: Retinal transplantation of photoreceptors results in donor–host cytoplasmic exchange
Source: Nat Commun. 2016 Oct 4;7:13028. doi: 10.1038/ncomms13028 (PMC5059459; doi:10.1038/ncomms13028)
Supplement: Supplementary Information — Supplementary Figures 1-4 [file ncomms13028-s1.pdf]

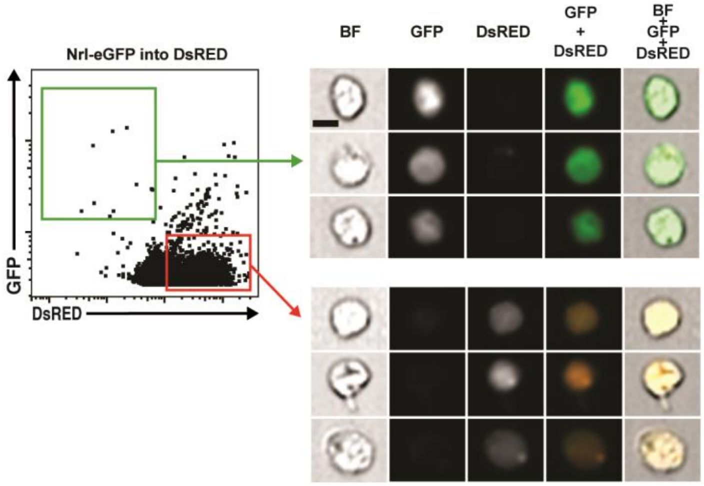

Supplementary Figure 1: Single GFP$^+$ or DsRED$^+$ expressing cells in transplanted retinas. Exemplary images of events from GFP$^+$ (green gate) and DsRED$^+$ (red gate) population obtained by imaging flow cytometry. Scale bar: 5µm. BF: brightfield.

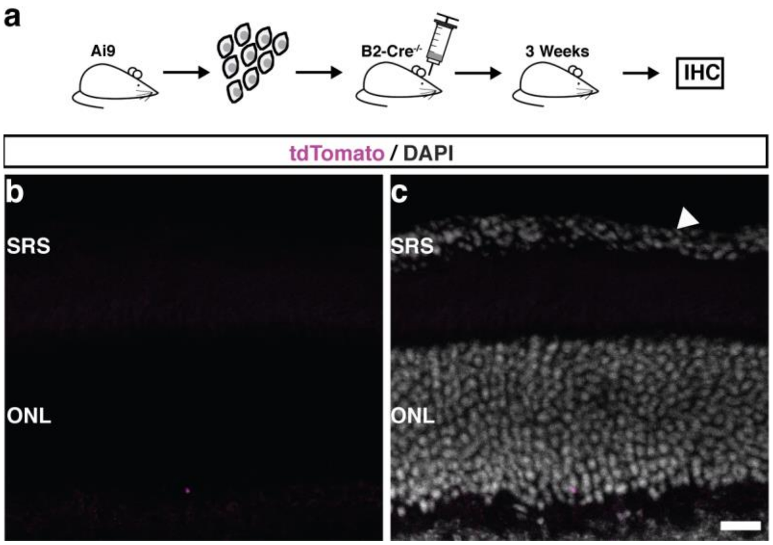

Supplementary Figure 2: Transplantation of Ai9 donor photoreceptors into B2-Cre$^{-/-}$. (**a**) Schematic representation of the control experiment and analysis of host retinas. (**b, c**) Three weeks after transplantation of Ai9 photoreceptors into B2-Cre$^{-/-}$ recipients (n=4), neither donor (arrowhead in **c**) nor host photoreceptors express tdTomato. Scale bar: 20µm. SRS: sub-retinal space; ONL: outer nuclear layer; IHC: immunohistochemistry.

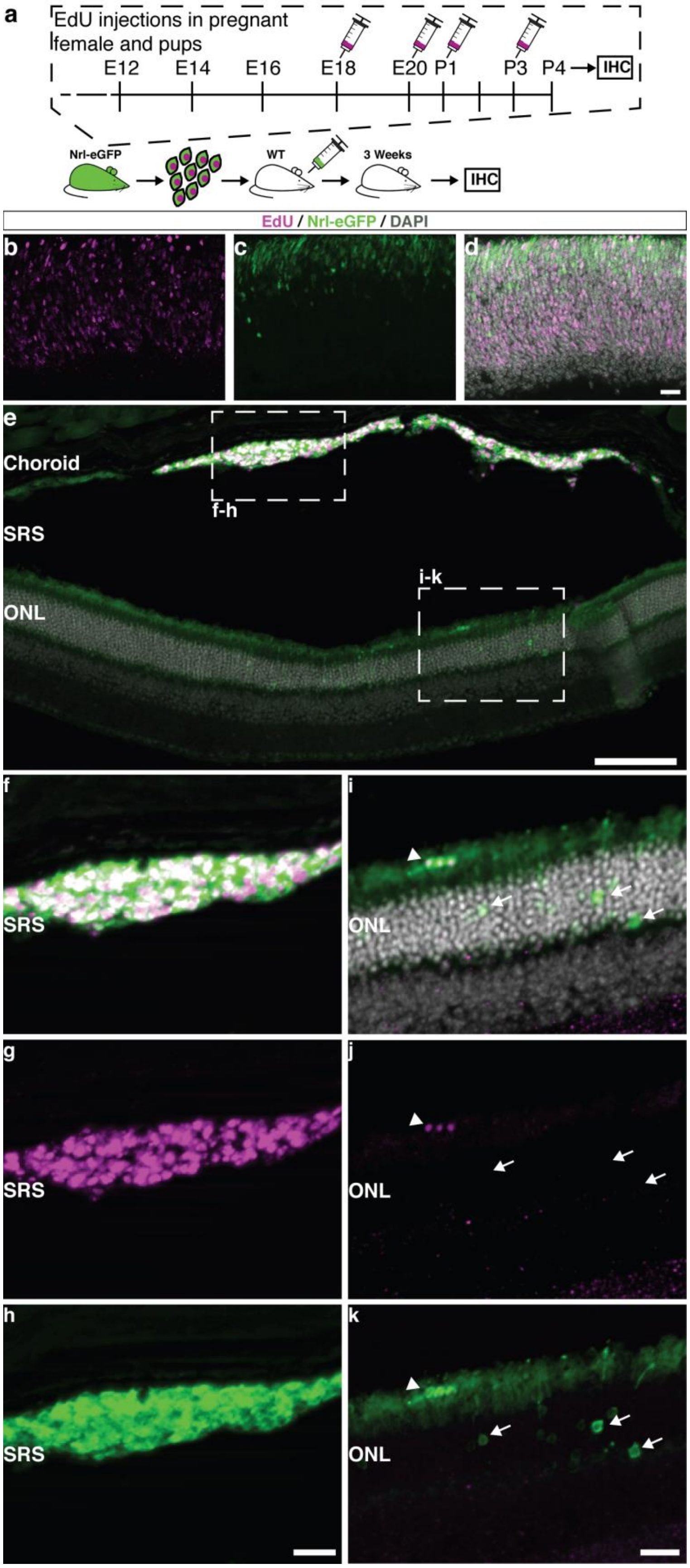
a
EdU injections in pregnant female and pups
E12
E14
E16
E18
E20
P1
P3
P4
IHC
Nrl-eGFP
WT
3 Weeks
IHC
EdU / Nrl-eGFP / DAPI
b
c
d
e
Choroid
f-h
SRS
i-k
ONL
f
SRS
i
ONL
g
SRS
j
ONL
h
SRS
k
ONL

Supplementary Figure 3: Transplantation of EdU$^+$/eGFP$^+$ donor photoreceptors. (**a**) Experimental outline: photoreceptors were isolated from Nrl-eGFP donors following four consecutive EdU injections during development and transplanted into wild-type hosts (n=3). (**b-d**) Postnatal day (P) 4 Nrl-eGFP retinas display EdU positivity throughout the retina after injections at embryonic day (E) 18, E20, P1 and P3. However, not all eGFP$^+$ photoreceptors show double labelling for EdU. (**e**) Overview of grafted area (increased space between graft and host ONL results from a histological (cutting) artifact). (**f-h**; magnification of the boxed area in **e**) Donor photoreceptors located in the sub-retinal space display co-localization for EdU and eGFP. (**i-k**; magnification of boxed area in **e**) GFP-positive cells located in the host's ONL lack EdU labelling (arrows) indicative for transfer of plasma content but not nuclei from donor to host cells. The arrowhead points to three eGFP$^+$/EdU$^+$ cells attached but not integrated into the ONL. Scale bars in (**b-d**), (**f-h**) and (**i-k**): 20µm, (**e**): 100µm. EdU: 5-ethynyl-2'-deoxyuridine; SRS: sub-retinal space; ONL: outer nuclear layer.

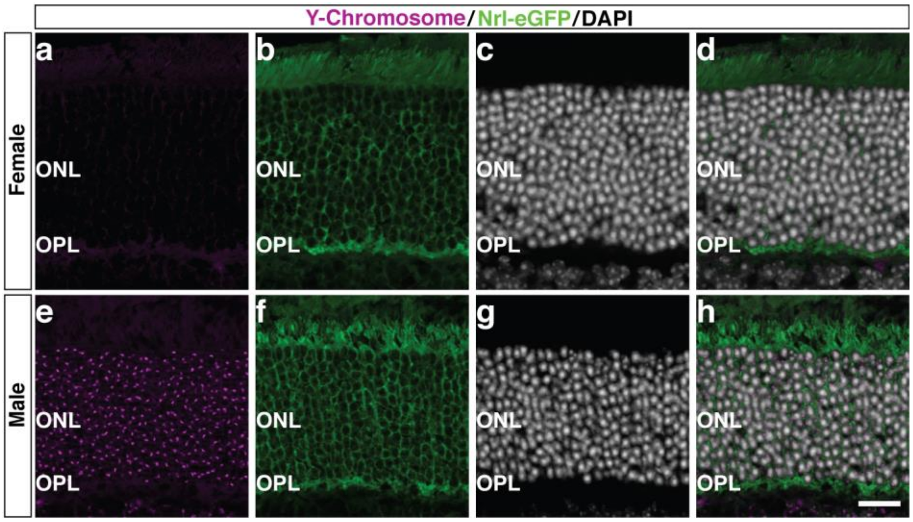

Supplementary Figure 4: Controls for Y-chromosome FISH. Adult Nrl-eGFP retinas from male and female mice were analysed using chromosomal fluorescent *in situ* hybridization (Y-chromosome FISH) and immunohistochemistry for eGFP. (**a-d**) In the female retina no Y-chromosome signal was detected. (**e-h**) In the male retina the presence of a Y-chromosome was detected in all cell nuclei of the ONL. Scale bar: 20µm. ONL: outer nuclear layer; OPL: outer plexiform layer.
